# The Eye in the Neck: Removal of a Sewing Needle from the Posterior Pharyngeal Wall

**DOI:** 10.1155/2010/608343

**Published:** 2010-12-08

**Authors:** Samit N. Unadkat, Rishi Talwar, Neil Tolley

**Affiliations:** Department of ENT Surgery, St Mary's Hospital, London W2 1NY, UK

## Abstract

Foreign body ingestion is a frequent presenting complaint to most emergency departments but the finding of a sewing needle in the posterior pharynx particularly is a rare finding. We report a case of a male patient with a sewing needle lodged in the posterior pharynx despite a history suggestive of chicken bone ingestion, absent clinical features, and negative flexible endoscopic examination. The needle was only identified through cervical spine radiographs. Even subsequent pharyngoscopy, laryngoscopy, and upper oesophagoscopy all proved to be unremarkable with the patient eventually requiring a left neck exploration to remove the needle. The case outlines the importance of simple radiography in suspected foreign body ingestion, even though clinical and endoscopic findings may be unremarkable.

## 1. Introduction

The use of cervical spine radiographs in the investigation of suspected foreign body ingestion remains a contested issue amongst ENT surgeons, radiologists, and accident and emergency doctors alike. However, no clear consensus has been reached with many physicians and surgeons still advocating discharge from hospital without cervical spine radiographs despite a positive history for foreign body ingestion but negative findings on flexible endoscopy and absent clinical signs.

We report an unusual case of a sewing needle stuck in the posterior pharynx following an unusual preceding history. To our knowledge, this is the first reported English-language case involving the posterior pharyngeal wall. This unusual case study is relevant to doctors across different medical and surgical specialities. It illustrates the potential consequences of a failure to correctly identify foreign body ingestion and the importance of imaging when there is a history of foreign body ingestion, despite the absence of specific clinical signs.

## 2. Case

A 49-year-old Nigerian male presented to the emergency department of St Mary's Hospital, London, after experiencing sudden left-sided throat pain while eating chicken. There was mild dysphagia and odynophagia but no dyspnoea. His past medical history was unremarkable.

On examination, the patient was apyrexial but distinctly hypertensive (190/112 mmHg). Observations were otherwise within normal limits. He had a full range of neck movements, and there was no obvious external neck swelling palpable. However, some tenderness was elicited just lateral to the thyroid cartilage in the left anterior triangle.

Flexible nasendoscopy and laryngoscopy revealed only mild erythema over the posterior wall but no foreign body was seen. Specifically, there was no pooling of saliva in the piriform fossae, and the patient was still able to eat and drink.

Despite the relatively unremarkable examination, postero-anterior (PA) and lateral soft tissue cervical spine radiographs were requested for completeness. To our surprise, these revealed a sewing needle, measuring 34.5 mm, lodged in the soft tissues of the posterior pharyngeal wall between C4–C6. The eye of the needle was clearly visible on magnification of the images.

A CT scan was ordered to further delineate the needle's location, in view of its apparent proximity to large vessels. The patient was placed nil by mouth and given intravenous fluids. He was additionally reviewed by a cardiologist for his hypertension, with an echocardiogram and ECG revealing left ventricular hypertrophy. The patient was thus commenced on nifedipine.

The patient was taken to theatre and underwent direct pharyngoscopy, laryngoscopy, and upper oesophagoscopy. These were all unremarkable. Consequently, left neck exploration and foreign body removal were undertaken. A left-sided skin crease neck incision was made, and dissection continued identifying the left internal jugular vein and carotid artery in the process. With the pharynx exposed, the needle was located penetrating the lateral part of the posterior pharyngeal wall and was easily extracted. There was no evidence of a residual perforation once removed. The patient's postoperative course was uneventful, commencing on sterile water the following day and discharged with a course of prophylactic antibiotics on the second postoperative day, eating and drinking normally.

## 3. Discussion

Foreign bodies lodged in the pharynx are not uncommon findings in the accident and emergency setting. Frequently, these can be attributed to fish bone ingestion [1]. Patients that have a suspected swallowed foreign body tend to present with mild throat discomfort and dysphagia, progressing to odynophagia, dyspnoea, and surgical emphysema in more severe cases.

Our particular case warrants analysis for a number of reasons. The first is the unusual preceding history. To date, it remains unclear as to how the needle became lodged in the posterior pharyngeal wall. Given the history, one might ascribe the symptoms of the foreign body retention to the chicken bones from the meal consumed. However, it is imperative that assumptions about the nature of the foreign body are not made.

Bones are commonly found by flexible endoscopy and depending on their location and can often be removed without the need for general anaesthesia [1, 2]. In this case, the flexible endoscopy merely revealed mild erythema but no obvious foreign body. The needle was only identified on posteroanterior and lateral soft tissue cervical spine radiographs. Chicken and fish bones can often be missed on plain films especially when the bone is lodged in an area of high soft tissue overlap [3], and sometimes they are radiolucent. Hence, if clinical findings are negative, there is a temptation to discharge the patient, especially if their symptoms are mild and nonspecific but the consequences of missing a foreign body are potentially life-threatening. (Of note, the effective radiation doses in anteroposterior and lateral cervical spine radiographs are 0.12 and 0.02 mSv, resp. This compares favourably to a routine chest radiograph, with a radiation dose of between 0.06 and 0.25 mSv [4]). 

Complications of foreign body retention are numerous and depend upon the nature of the foreign body involved, its location, and ultimately the duration of impaction [5, 6]. If swift action is not taken, one potentially risks oesophageal perforation that can lead to fistula and abscess formation. According to one study, the sharper the foreign body, the earlier the risk of perforation [7]. Rare studies have even shown swallowed metal pins migrating to the superior mediastinum, ultimately requiring a median sternotomy for retrieval.

Various ENT and emergency department studies have investigated the necessity for radiographic evaluation of suspected foreign body ingestion in the absence of obvious clinical signs. One such study by Marais et al. (1995) noted that radiography only correctly identified 38.3% of all foreign bodies with over one quarter of the patient population having a false positive diagnosis [8]. This was further backed up by Evans et al. (1992), who stated that plain radiography had a sensitivity of just 25.3% and that routine radiography for suspected fish bone impaction, as was the case in our patient, ought to be abandoned [9]. Neither study though takes into consideration variability between the interpreting clinician. 

Interestingly, Karnwal et al. (2008) looked at just this point and found that emergency department and ENT doctors missed almost 80% and 67% of all positive findings on radiography, with lateral neck X-rays helping in over 50% of all patients with foreign body ingestion [10]. They advocate greater radiology training to all junior ENT and emergency department doctors in recognising foreign bodies from lateral neck radiographs.

In conclusion, a high index of suspicion must always remain in any patient presenting acutely with a history of foreign body ingestion, even in the absence of specific clinical signs. The minimal radiation exposure from cervical spine radiographs is an acceptable risk but the consequences of incorrectly discharging patients are potentially life-threatening. If there is no obvious foreign body visible on flexible endoscopy, we recommend imaging initially with radiographs, subsequently with CT, and if necessary, endoscopy under a general anaesthetic.

## Figures and Tables

**Figure 1:**
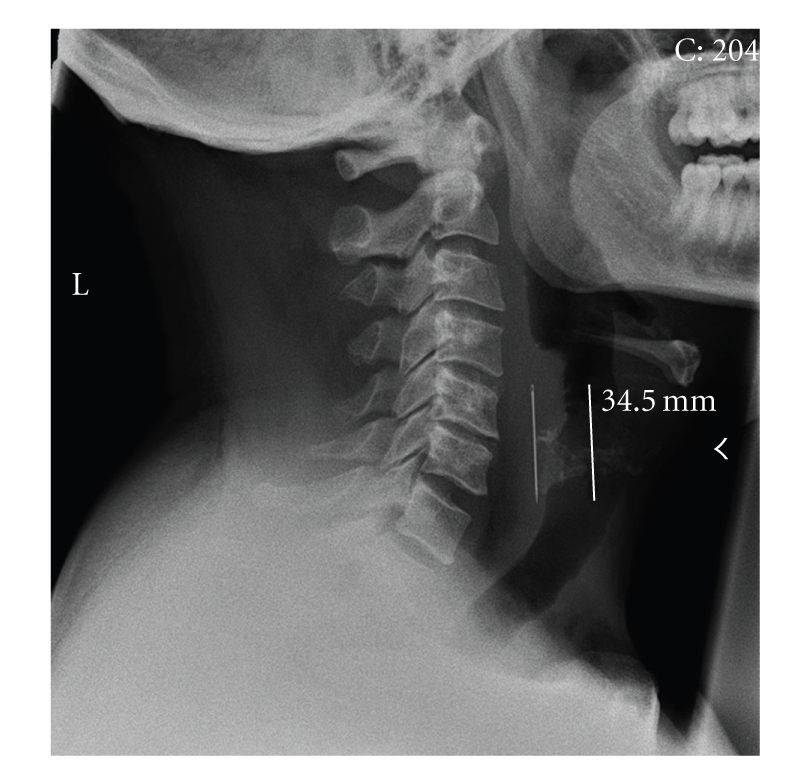
Lateral cervical spine radiograph.

**Figure 2:**
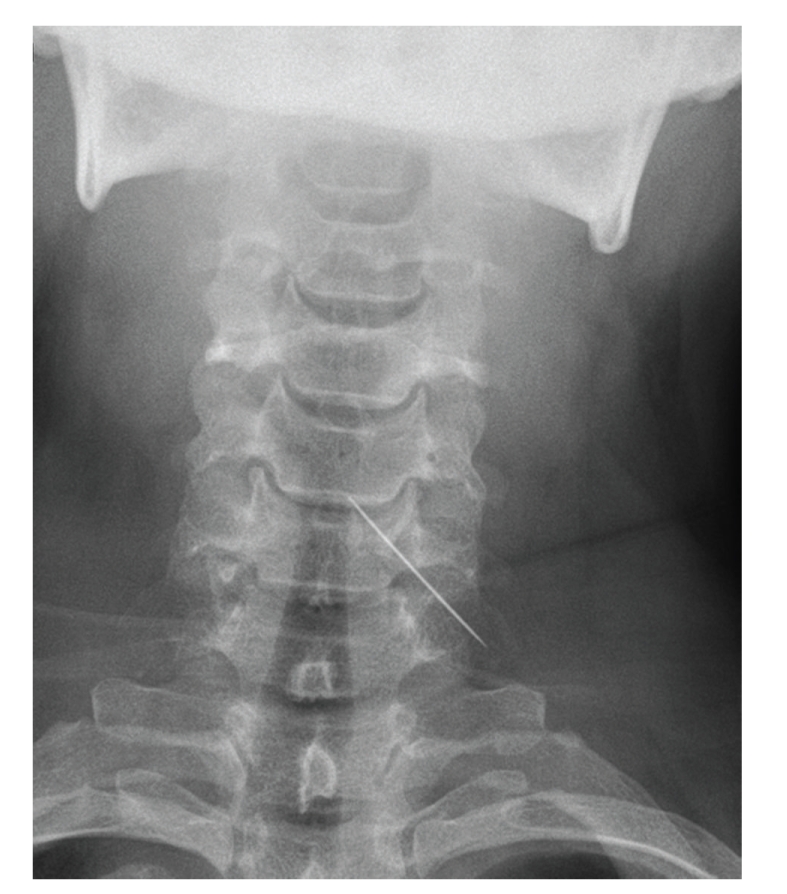
Postero-anterior cervical spine radiograph.
